# Clinical Responses to Prostate-specific Membrane Antigen Radioguided Salvage Lymphadenectomy for Prostate Cancer Recurrence: Results from a Prospective Exploratory Trial

**DOI:** 10.1016/j.euros.2024.09.004

**Published:** 2024-10-15

**Authors:** Adam B. Weiner, Zachary Ells, Catherine Meyer, Magnus Dahlbom, David Sennung, Deepu Varughese, Vinicius B. Ludwig, Giuseppe Carlucci, Raeven Grant, Johannes Czernin, Jeremie Calais, Robert E. Reiter

**Affiliations:** a Department of Urology, David Geffen School of Medicine, University of California-Los Angeles, Los Angeles, CA, USA; b Institute for Precision Health, University of California-Los Angeles, Los Angeles, CA, USA; c Ahmanson Translational Theranostics Division, Department of Molecular and Medical Pharmacology, David Geffen School of Medicine, University of California-Los Angeles, Los Angeles, CA, USA; d Jonsson Comprehensive Cancer Center, University of California-Los Angeles, Los Angeles, CA, USA

**Keywords:** GA-68 PSMA-11, Lymph node excision/methods, Positron emission tomography, Prostatic neoplasms/surgery, Salvage therapy, Tomography, Emission computed, Single photon

## Abstract

**Background and objective:**

Prostate-specific membrane antigen (PSMA) radioguided salvage pelvic lymph node dissection (S-PLND) has emerged as a feasible treatment option for prostate cancer recurrence following initial surgery. This study aims to evaluate the feasibility and short-term outcomes of PSMA radioguided S-PLND.

**Methods:**

From a prospective trial of ^99m^Tc-PSMA-I&S followed by PSMA radioguided robotic surgery, we evaluated patients treated for node-only recurrence following radical therapy. The primary outcome was serum prostate-specific antigen (PSA) response 3 mo after surgery.

**Key findings and limitations:**

Among 14 patients (enrolled from June 2021 to June 2023), the median age was 65 yr. One patient had undergone primary whole gland ultrasound ablation, while the rest received prior prostatectomy. The median (interquartile range) time from primary treatment to PSMA positron emission tomography (PET) was 4.1 (2.9–8.3) yr, and 21 total pelvic targets were noted on PSMA PET: one in eight patients (67%), two in five patients (29%), and three in one patient (7%). Targets were successfully detected intraoperatively and removed in 13/14 (93%) patients. Cancer was noted on histopathology in 90% (19/21) of PSMA PET targets, 94% (17/18) of single-photon emission computed tomography targets, and 82% (14/17) of gamma probe targets. There were no adverse effects due to the radiotracer, and there were no complications after surgery. PSA at 3 mo was <0.2 ng/ml in two (14%) patients, and a ≥50% decline was noted in five (36%) patients. After a mean follow-up of 8.3 mo, the median time to next treatment was 11.7 mo, which was noted in nine patients.

**Conclusions and clinical implications:**

PSMA radioguided S-PLND is feasible and safe. However, the clinical role and the honing of technique and patient selection will be required in prospective studies.

**Patient summary:**

In 14 patients who had prostate cancer recurrence after their initial treatment, performing surgery using radioactive tags to location is possible. However, futures studies are still needed to improve the technique.

## Take Home Message

Prostate-specific membrane antigen radioguided salvage pelvic lymph node dissection is feasible but requires continued honing of the technique to optimize oncologic outcomes.

## 1 Introduction

Prostate cancer (PCa) recurrence following initial surgery is commonly managed with salvage pelvic radiotherapy or long-term systemic hormonal therapies [1]. However, metastasis-directed therapy (MDT) for patients with minimal metastatic disease (oligometastatic) may delay or minimize treatment-related morbidities [2], [3], [4], [5]. Additionally, prostate-specific membrane antigen (PSMA) positron emission tomography (PET) can help localize recurrent disease and potentially improve MDT efficacy [5], [6]. Most trials investigating MDT have leveraged radiotherapy, but salvage pelvic lymph node dissection (S-PLND) offers another approach [7]. Prostate-specific antigen (PSA) response rates after S-PLND, however, have varied from 0% to >80% in studies limited by the use of imaging other than PSMA PET and malignant histologic findings in as few as 22% of patients [7]. In a retrospective study of 33 patients with recurrence following primary surgery and PSMA PET before and after surgery [8], 15/33 (45%) had persistent lesions that were visible before surgery, highlighting the difficulty for surgeons to locate small pelvic lymph node targets intraoperatively using a cognitive interpretation of imaging findings.

In this context, PSMA radioguided S-PLND has emerged as a promising treatment option [9], [10], [11], [12]. However, studies of PSMA radioguided S-PLND have reported comparable oncologic outcomes to those of S-PLND without radioguidance [11], [12], [13]. Hence, further refinement to optimize oncologic outcomes is required. This report presents initial oncologic outcomes from a prospective trial of ^99m^Tc-PSMA-I&S radioguided surgery.

## 2 Patients and methods

### 2.1 Patients and trial design

This was a prospective exploratory biodistribution trial conducted under the Radioactive Drug Research Committee Program (title 21 of Code of Federal Regulations, section 361.1). The primary objective of the trial was to define the biodistribution of ^99m^Tc-PSMA-I&S in normal and malignant tissues of patients with PCa with histopathology validation. The trial was self-funded, approved by the University of California-Los Angeles (UCLA) Institutional Review Board (IRB#20-002256) and registered on ClinicalTrials.gov (NCT04857502). All patients provided oral and written informed consent. Patients with primary or recurrent PCa with evidence of PSMA PET visible lymph node metastasis considered for pelvic lymph node dissection (PLND) were eligible. Any PSMA PET tracer was allowed. Patients who started any PCa treatment between study enrollment and surgery, or those with technically inaccessible nodal location were excluded.

Here, we report a preliminary post hoc analysis of the oncologic outcomes of patients who underwent radioguided S-PLND (*n* = 20). The protocolized trial primary outcome was the biodistribution of the radiopharmaceutical used (trial protocol in the Supplementary material). This outcome will be presented upon complete trial recruitment (*n* = 30). Clinical outcomes were not predefined and were assessed retrospectively for this work. Patients with planned prostatectomy at the time of this trial were eligible for the trial but were excluded from this secondary analysis.

### 2.2 Synthesis of ^99m^Tc-PSMA-I&S

The ^99m^Tc-PSMA-I&S radiopharmaceutical was prepared in the UCLA Biomedical Cyclotron Facility, employing pharmaceutical-grade materials and Good Manufacturing Practice following *United States Pharmacopeia*, chapter 28. A commercially available ^99m^Technetium generator and reagents were used; 40 μg of PSMA-I&S precursor was used per dose. The radiopharmaceutical ^99m^Tc-PSMA-I&S was manufactured with a radiochemical purity of ≥90%. The target injected activity was 650–750 MBq (17–20 mCi) based on prior report for ^99m^Tc-PSMA-I&S single-photon emission computed tomography (SPECT) imaging [14].

### 2.3 SPECT imaging with ^99m^Tc-PSMA-I&S

Patients underwent ^99m^Tc-PSMA-I&S tracer administration the day prior to planned S-PLND. SPECT/computed tomography (CT) image acquisition was performed at 4 h after the injection. This provided visual confirmation of lesion(s) uptake to aid surgical planning. SPECT/CT acquisition was completed at 4 h using the dual-detector Symbia Intevo (Siemens Healthineers, Erlangen, Germany). The following parameters for the SPECT were used: noncircular orbit, step and shoot, 120 views, 20 s per view, and 256 × 256 matrix size. For the CT, 130 kVp, care dose 70 mAs, 5-mm slice, and 0.75 pitch were used. Reconstruction was carried out on the Siemens system using the Flash3D, eight iterations, six subsets, and 5-mm Gaussian filter.

### 2.4 Surgical intervention

**Fig. 1:**
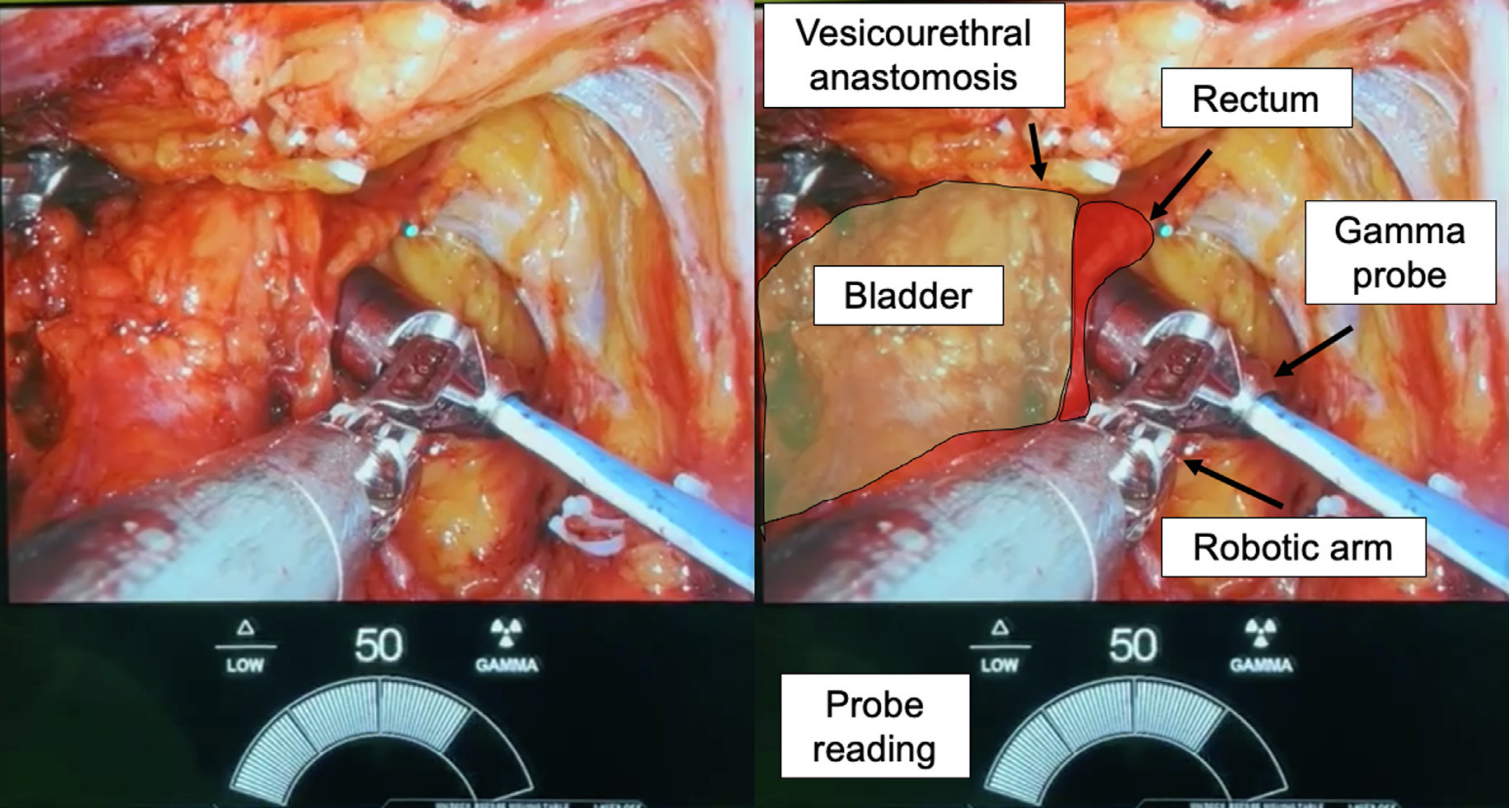
Intraoperative image of surgeon using a drop-in gamma probe. Image of surgeon’s view on robot console without (left) and with (right) labels. Probe reading was suggestive of a positive target in a patient with a known right perirectal target on PSMA PET. PET = positron emission tomography; PSMA = prostate-specific membrane antigen.

**Fig. 2:**
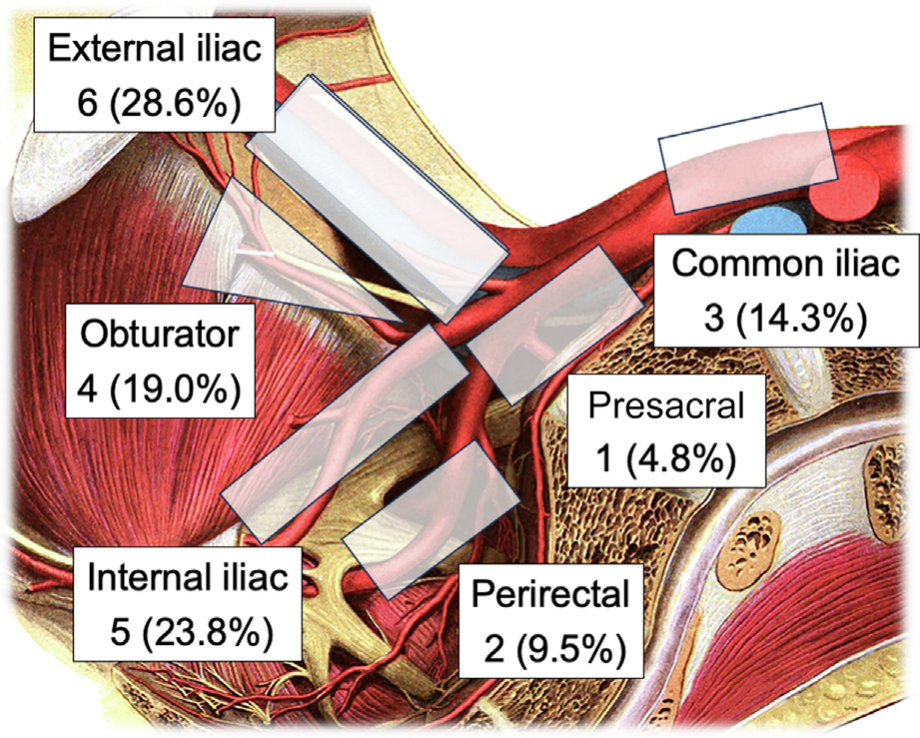
Location of node targets on preoperative PSMA PET imaging. PET = positron emission tomography; PSMA = prostate-specific membrane antigen. (License information for open access figure: “Sobotta 1909 fig.568 - Blood vessels and nervus of the pelvic wall - no labels” at AnatomyTOOL.org by Johannes Sobotta and dream_studio3, license: Creative Commons Attribution-ShareAlike.)

The patients then underwent multiport robotic-assisted laparoscopic S-PLND the day following the tracer administration (+16–22 h). Target lymph node identification was performed using a drop-in gamma probe that was inserted through a 12-mm assistant port. The first two surgeries used a custom variation of the Nodeseeker probe (IntraMedical Imaging, Hawthorne, CA, USA), while all other surgeries used the SENSEI drop-in gamma probe (Lightpoint, a Telix company, London, UK; Fig. 1). Although no threshold for drop-in gamma probe positivity was noted as part of the trial protocol, a cutoff of ≥40 counts per second was typically used, and background from the bladder and bowel was accounted for on an individual patient basis. The extent of lymph node dissection was up to surgeon discretion, who decided whether to remove tissue from the single affected area or from multiple packets. The extent of S-PLND was determined based on operative reports. A dissection was considered extensive if the surgeon reported removing tissue from at least three regions, as noted in Figure 2. All others were considered focal. Ex vivo measurements of the resected tissue were performed on a table in the operating room to ensure that radio-labeled tissue was contained in the excised tissue. The removed tissue was assessed within our institution’s pathology core by a trained genitourinary pathologist to identify PCa.

### 2.5 Outcomes

The primary outcome of this secondary analysis was a serum PSA level of ≤0.2 ng/ml 3 mo after PLND. Other outcomes included a 50% decline in PSA (PSA50) 3 mo after PLND, node-positive disease on a pathologic analysis by lesion from PSMA PET, and time to additional salvage treatment. PSA monitoring and use of additional PCa treatment were all part of routine care, and were assessed by a retrospective chart review.

### 2.6 Statistical analysis

Time to additional treatment was estimated using the Kaplan-Meier method. Descriptive statistics were also reported for patients. R version 4.3.2 (R Foundation for Statistical Computing, Vienna, Austria) was used.

## 3 Results

### 3.1 Patient characteristics

A total of 20 patients were enrolled in the trial and underwent initial tracer administration and SPECT/CT (from June 2021 to June 2023). Patient #05 had cardiac arrhythmias in the preoperative evaluation and thus never underwent S-PLND. Five patients were excluded from this secondary analysis since they underwent prostatectomy at the time of PLND. Therefore, 14 patients were assessed (Table 1). The median patient age was 65 yr (interquartile range [IQR]: 59–72). Nine (64%) patients had a history of grade group 4–5 disease. Patient #13 underwent prior whole gland high-intensity focused ultrasound, while the rest underwent prostatectomy. Patient #07 was managed with salvage intermittent androgen deprivation therapy (ADT) after a biochemical recurrence following prostatectomy in 1999 and had castration-resistant PCa (PSA 3.1 ng/ml) at the time of PSMA PET.

**Table 1:** Patient characteristics

Characteristic	Median (IQR)/*n* (%)
Total	14 (100)
Time between primary treatment and PSMA PET (yr)	4.1 (2.9–8.3)
Time between PSMA PET and ^99m^Tc-PSMA-I&S injection (d)	87 (79–142)
Injected activity of ^99m^Tc-PSMA-I&S (MBq)	722 (692–730)
Time between ^99m^Tc-PSMA-I&S injection and surgery (h)	19.2 (18.5–19.5)
Age (yr)	65 (59–72)
Grade group	
2	2 (14)
3	3 (21)
4	3 (21)
5	6 (43)
pT stage	
2	6 (43)
3	6 (43)
Unknown	2 (14)
pN stage	
0/x	11 (79)
1	1 (7)
Unknown	2 (14)
PET isotope	
Gallium-68 PSMA-11	10 (71)
Piflufolastat F-18	4 (29)
PSA at time of PET (ng/ml)	1.03 (0.48–2.80)
Lesions on PET	
1	8 (57)
2	5 (36)
3	1 (7)
Target SUVmax	4.8 (3.2–6.7)
Target size (cm)	0.23 (0.18–0.30)
Lymph nodes removed at surgery	2 (1–2)

IQR = interquartile range; PET = positron emission tomography; PSA = prostate-specific antigen; PSMA = prostate-specific membrane antigen; SUVmax = maximum standardized uptake value.

The median serum PSA prior to S-PLND was 1.03 ng/ml (IQR: 0.48–2.80; Table 1), and the median time from primary treatment to PSMA PET was 4.1 yr (IQR: 2.9–8.3). The PSMA PET tracer employed was gallium-68-PSMA-11 for ten patients (71%) and F18-DCFPyL for four patients (29%). Most patients had only one target node on PSMA PET (eight patients, 67%), five (36%) had two targets, and one had three targets, for a total of 21 target regions (mean: 1.5). Most target regions were located either in the external iliac (six patients, 29%) or in the internal iliac (five patients, 23.8%) space (Fig. 2). The median target standardized uptake value was 4.8 (IQR: 3.2–6.7) and target size was 0.23 cm (IQR: 0.18–0.30).

### 3.2 Administration of Tc^99m^-PSMA-I&S and SPECT/CT

The median time interval between PSMA PET and ^99m^Tc-PSMA-I&S injection was 87 d (IQR: 79–142). The median injected activity of ^99m^Tc-PSMA-I&S was 722 MBq (IQR: 692–730). There were no adverse effects from the tracer administration. SPECT/CT acquisition was performed 4 h after tracer administration. Of the 21 targets seen on PSMA PET, 18 (86%) were visible on the SPECT images (Supplementary Table 1).

### 3.3 Perioperative summary and pathology

Surgery was performed a median of 19.2 h (IQR: 18.5–19.5) after the ^99m^Tc-PSMA-I&S injection (Table 1). The median operative time was 98 min (IQR: 84–103.5). Of the 21 targets noted on PSMA PET, 17 could be detected with the intraoperative drop-in gamma probe in the expected location. Most patients received extensive S-PLND defined as at least three regions (Fig. 2) of tissue removal, as noted by operative reports (nine of 14, 64%). A total of nine (64%) patients stayed one night in the hospital, while the rest were discharged the same day of surgery. There were no 90-d Clavien-Dindo complications or readmissions.

### 3.4 Pathology and clinical response

Tissue from targets on PSMA PET were removed in 13/14 (93%) patients, and carcinoma on final histopathology was detected in all these patients. A median of two nodes were removed from each patient. Tissue from one perirectal target in one patient was not resected because of safety concerns with localization intraoperatively related to scar tissue from salvage radiotherapy. An additional patient with common iliac targets on PSMA PET had positive targets superior into the retroperitoneum on the drop-in gamma probe, which was not pursued with dissection based on surgeon discretion. Carcinoma was noted on histopathology in 90% (19/21) of PSMA PET targets, 94% (17/18) of SPECT targets, and 82% (14/17) of drop-in gamma probe targets (Supplementary Table 1).

**Fig. 3:**
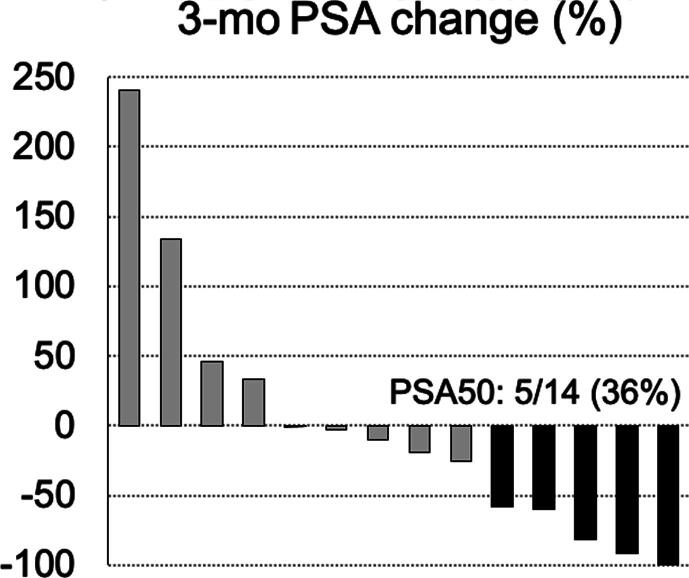
Waterfall plot of serum prostate-specific antigen change at 3 mo following salvage pelvic lymph node dissection. A total of two patients noted a 3-mo PSA level of ≤0.2 ng/ml. Black bars indicate patients who had ≥50% decline in serum PSA. PSA = prostate-specific antigen; PSA50 = ≥50% decline in serum PSA.

**Fig. 4:**
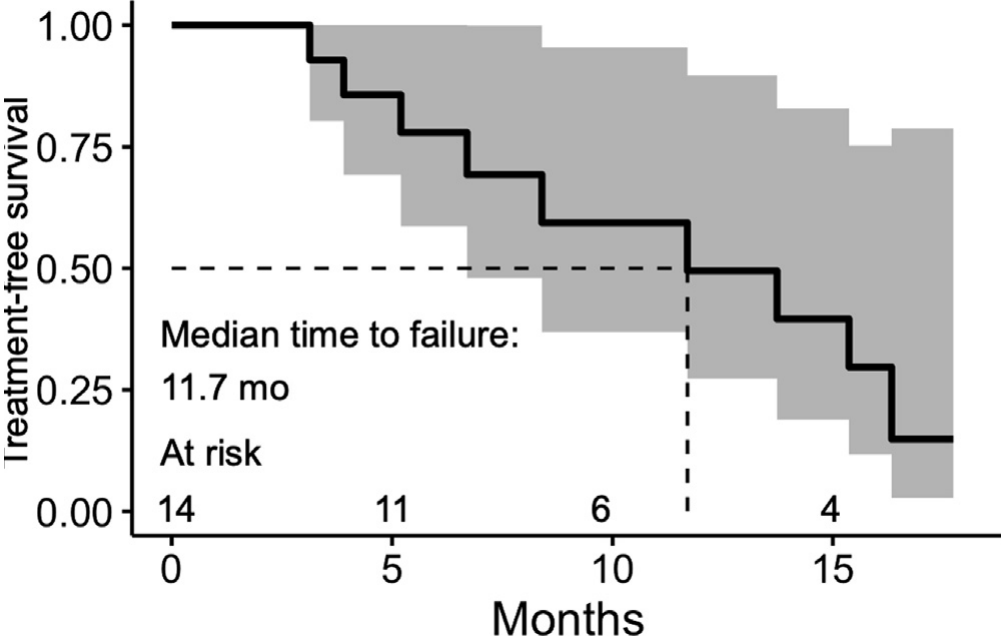
Kaplan-Meier estimates for time to next treatment after salvage pelvic lymph node dissection. Shading indicates 95% confidence intervals.

A total of two (14%) patients had a PSA value of ≤0.2 ng/ml 3 mo after S-PLND (Fig. 3). Both patients had only single PSMA PET targets and underwent extensive S-PLND. One patient had grade group 5 disease, while the other had grade group 2 disease at prior radical prostatectomy. PSA50 was noted in five (33%) patients: four had only one PSMA PET target and one had two. At a mean follow-up time of 8.3 mo, nine (64%) patients have received salvage radiotherapy or systemic therapy (Fig. 4). The median time to additional treatment was 11.7 mo. Of the nine additional treatments, three were radiotherapy MDT with hormone therapy, three were radiotherapy MDT with PSMA-targeted radioligand therapy on a clinical trial, two were radiotherapy MDT alone (one on a clinical trial), and one was hormone therapy alone.

## 4 Discussion

The advent of PSMA PET has enabled better localization of recurrent PCa. In this prospective trial, we assessed the feasibility and clinical outcomes of ^99m^Tc-PSMA-I&S radioguided S-PLND in patients with limited nodal recurrence on PSMA PET in the pelvis. The data from 14 patients suggest that this approach is feasible, but still requires optimization and patient selection.

In a retrospective series of 163 patients who underwent S-PLND, over half received ADT within 5 yr and about two-thirds had complete PSA responses [13]. Similarly, in a cohort of 121 patients who had PSMA radioguided S-PLND, 66% experienced a complete PSA response (PSA <0.02 ng/ml) [10], [13]. Radioguided surgery increased the metastatic tissue yield from 79% to 99%, with better outcomes seen in patients with single lesions on PSMA PET and lower PSA levels before surgery. Accordingly, both patients with complete PSA responses in the current study had only single targets on PSMA PET. This suggests that multiple PSMA PET targets suggest micrometastatic disease outside of what is visible on PET.

It is also important to acknowledge that the sensitivity of PSMA PET for detecting pelvic nodal disease is about 0.4 and as low as 0.29 for nodes small than 1 cm [15]. Sensitivity also tends to increase for patients with higher-grade primary disease [16]. In this work, the median target size on PET was 0.23 cm, and future work should evaluate the efficacy of radioguided S-PLND based on PSMA PET target size and primary grade group.

One other group reported a prospective initial evaluation of PSMA radioguided S-PLND in 20 patients who had pelvic recurrence following primary prostatectomy or radiotherapy [12]. Of the 21 PSMA-avid targets noted on PET in these patients, 19 (90%) were localized using a drop-in probe during surgery. However, similar to our findings, a limited number of patients achieved a PSA complete response (<0.2 ng/ml; 4/18, 22%). Other series on patients undergoing radioguided PLND during prostatectomy [14], [17], [18], [19], [20] or repeat S-PLND [21] show similar results. Notably, while comparative work suggests that radioguided surgery can improve PSA response rates compared with standard S-PLND [22], these results collectively suggest that more efforts are needed to hone the approach and define, for instance, standardized complete resection and assessment of surgical margins. Horn et al [10] performed a phase 2 trial with 121 patients undergoing salvage radioguided surgery for recurrent PCa. In their work, surgeons performed extensive templated dissections based on PET findings. Complete biochemical responses were noted in 66% of patients, suggesting that extensive dissections might help optimize oncologic outcomes. The current study was limited by the lack of a defined surgical template and use of a limited template. The ideal approach also requires optimization. Prior works suggest that radioguidance during both open and robotic surgery can help identify nodal metastases outside of an extended PLND template [19], [20]. Additionally, a prior cohort study assessed 22 patients who underwent S-PLND following PSMA PET [23]. Despite not using an intraoperative probe for radioguidance, the authors showed that eight (36%) patients achieved a PSA complete response. The current trial did not define dissection extent, and thus not every patient received a template based on PSMA PET targets. Thus, surgeons could decide whether they wanted to remove tissue from just the affected area or packets in proximity. This would add heterogeneity to the trial’s surgical intervention. Forthcoming trials should define templates similarly to that of Horn et al [10], to homogenize S-PLND techniques and define the value of the additional steps and resources needed for radioguidance.

One way to improve complete resection of both primary and nodal disease includes the use of a fluorescent agent [24]. Nguyen et al [25] used a PSMA-targeting agent in 24 patients with high-risk PCa undergoing prostatectomy with PLND. The agent with fluorescent light had a 97% negative predictive value for nodal metastases. Whether or not this approach could improve the rates of complete removal of pelvic metastatic disease, perhaps in combination with radioguidance, remains to be seen.

For patients undergoing S-PLND without radioguidance, inclusion of hormone therapy might improve oncologic outcomes [13]. The TRACE-II study will randomize 60 patients with pelvic nodal metastatic disease (fewer than three lesions), following primary definitive therapy, to 6 mo of ADT ± radioguided S-PLND [26]. It will assess progression-free survival at 2 yr to learn the benefits of radioguided surgery. Ultimately, additional prospective work, such as this, with oncologic outcomes will be required to determine whether MDT with radioguided S-PLND can help improve the natural history of oligometastatic PCa. The timing of this sort of intervention will also be crucial to balance with the fact that most patients can and will want to delay any intervention for biochemical recurrence—in particular those including ADT.

Limitations include small sample size, short follow-up, and a lack of robust oncologic outcomes, such as death from PCa. Additionally, no patients underwent pelvic magnetic resonance imaging, which may be superior to PSMA PET in detecting local recurrences [27]. The cohort was heterogeneous: one patient was treated previously with high-intensity focused ultrasound, one patient had castration-resistant PCa, and the number of PET targets varied. However, the conclusion remains—S-PLND with radioguidance is feasible and detects PET-positive lymph nodes, but requires refinements. Critical questions include establishment of a radioactive threshold to detect cancer, dissection templates, and the value of multimodality therapy. Additionally, while this was a prospective clinical trial, follow-up outcomes were retrospectively collected for this ad hoc analysis. This could potentially bias data collection and results. Finally, while surgeons in this trial generally felt that the probe was easy to use and helped find nodal disease that otherwise would have been difficult to find, surgeons were not surveyed formally on the ease of use of the drop-in gamma probe. While tissue was removed in nearly all patients in this trial, it is unclear how refinement in the instrument itself might affect results.

## 5 Conclusions

Our trial on radioguided S-PLND in recurrent PCa adds to the MDT surgery literature. While it is feasible and safe, complete PSA response is rare, indicating areas for future research focus.

  ***Author contributions*:** Adam B. Weiner had full access to all the data in the study and takes responsibility for the integrity of the data and the accuracy of the data analysis.

  *Study concept and design*: Weiner, Czernin, Calais, Reiter.

*Acquisition of data*: Weiner, Ells, Meyer, Dahlbom, Varughese, Ludwig, Carlucci.

*Analysis and interpretation of data*: Weiner, Ells, Reiter.

*Drafting of the manuscript*: All authors.

*Critical revision of the manuscript for important intellectual content*: All authors.

*Statistical analysis*: Weiner.

*Obtaining funding*: Weiner, Czernin, Calais, Reiter.

*Administrative, technical, or material support*: None.

*Supervision*: Calais, Reiter.

*Other*: None.

  ***Financial disclosures:*** Adam B. Weiner certifies that all conflicts of interest, including specific financial interests and relationships and affiliations relevant to the subject matter or materials discussed in the manuscript (eg, employment/affiliation, grants or funding, consultancies, honoraria, stock ownership or options, expert testimony, royalties, or patents filed, received, or pending), are the following: None.

  ***Funding/Support and role of the sponsor*:** Adam B. Weiner was supported by the Simon-Strauss Foundation, the UCLA Dr. Allen and Charlotte Ginsburg Fellowship in Precision Genomic Medicine, the Prostate Cancer Foundation Young Investigator Award (23YOUN21), and the Department of Defense (HT94252410589). This work was also supported by the UCLA NIH SPORE in Prostate Cancer (P50 CA09213). These funders had no specific role in the design and conduct of the study; collection, management, analysis, interpretation, preparation, and review of the data; and approval of the manuscript.

  ***Acknowledgments*:** We are thankful to all trial participants. This is an investigator-initiated trial. Telix Pharmaceuticals provided SENSEI drop-in gamma probe and related consumable. During the preparation of this work, the authors used artificial intelligence–assisted technologies in the writing process.

  ***Data sharing*:** All data generated or analyzed during this study are included in this published article.
