# Supplementary material for: Clinical Responses to Prostate-specific Membrane Antigen Radioguided Salvage Lymphadenectomy for Prostate Cancer Recurrence: Results from a Prospective Exploratory Trial
Source: Eur Urol Open Sci. 2024 Oct 15;70:36–42. doi: 10.1016/j.euros.2024.09.004 (PMC11525452; doi:10.1016/j.euros.2024.09.004)
Supplement: Supplementary Data 1 [file mmc1.docx]

**Supplementary Table 1:** Targets and correlation with histopathology

| Modality | Targets | Cancer on histopathology (%) |
| --- | --- | --- |
| PET | 21 | 19 (90) |
| SPECT | 18 | 17 (94) |
| Gamma probe | 17 | 14 (82) |

SPECT images were obtained four after tracer administration. Abbreviations: PET, positive emission tomography; SPECT, Single-photon emission computed tomography
